# Predicting the location of the axon initial segment using spike waveform analysis: simulations of retinal ganglion cell physiology

**DOI:** 10.1186/1471-2202-14-S1-P301

**Published:** 2013-07-08

**Authors:** Matias I Maturana, Raymond Wong, Tania Kameneva, Shaun L Cloherty, Michael R Ibbotson, Alex E Hadjinicolaou, David B Grayden, Anthony N Burkitt, Hamish Meffin, Brendan J O'Brien

**Affiliations:** 1 NeuroEngineering Laboratory, Dept. of Electrical Electronic Engineering, University of Melbourne, Australia; 2 Centre for Neural Engineering, University of Melbourne, Australia; 3 The National Vision Research Institute, Australian College of Optometry, Australia; 4 Eccles Institute of Neuroscience, Australian National University, Australia; 5 NICTA Victoria Research Lab, Australia; 6 Dept. of Optometry and Vision Science, University of Melbourne, Australia; 7 Bionics Institute, Australia

## 

There are 16 morphologically defined classes of rat retinal ganglion cells (RGCs). Most commonly, they are classified on the basis of several criteria including: soma size, dendritic field diameter, the dendritic branching pattern and the depth of stratification in the inner plexiform layer. Recently, it has also been shown that the intrinsic physiological properties of each rat RGC type vary enormously. Using multicompartment models of RGC types we investigated whether the location of the axon initial segment (AIS), the site of greatest sodium channel density and lowest voltage threshold, can be predicted by measurements of spike waveform made at the soma.

The action potential waveform in many neurons consists of several components, which can be determined by examining the first and second derivatives of the membrane potential. In this study, we focus on this technique as an objective method to analyze the action potential waveform for different morphological RGC types. In addition, we analyze the features of the phase plot, which shows the rate of change of the membrane potential against the membrane potential itself. Phase plot analysis allows the measurement of subtle differences in the action potential waveform such as the initial segment-soma/dendritic break (ISSD), which corresponds to the early rising phase of the action potential. When the recording is made at the soma, the presence of the ISSD in the phase plot indicates that a low threshold region (i.e. the AIS) is further away from the soma.

Rat RGCs were characterized electrophysiologically using standard whole cell patch clamp recording techniques. Data were acquired at 20 kHz using custom software developed in LabView (National Instruments). Spontaneous spikes and spikes evoked by just-threshold current were used for analysis. For each of the recordings, the amplitude and time of the trough between the peaks in the second-order derivatives were analyzed. After three dimensional confocal reconstruction of each recorded cell (Zeiss PASCAL) it was classified morphologically into one of the 16 predefined types. Multicompartment models of real retinal ganglion cells were constructed from 3D rendering confocal reconstructions and their physiology was simulated using the Hodgkin-Huxley formalism in the NEURON environment. Sodium channel density in the AIS and its distance from the soma were systematically varied and the effects on the phase plot analyzed.

Simulations showed that the further the AIS was from the soma, the more pronounced the ISSD break, resulting in a larger break with a deeper trough between the two peaks in the phase plot. This result allows us to predict the location of the AIS based on recordings of the impulse waveform. In addition, we found that the density of sodium channels in the AIS affects spike propagation into the soma. We observed that decreasing sodium conductance in the AIS, required two spikes to occur in the AIS in order to evoke a somatic spike. This was also observed experimentally, in particular in C4 cells. Further analysis of individual RGC spike waveforms demonstrated that certain RGC types could be reliably identified using their spike waveforms.

